# Subjective Health, Physical Activity, Body Image and School Wellbeing among Adolescents in South of Sweden

**DOI:** 10.3390/nursrep11040076

**Published:** 2021-10-21

**Authors:** Ann-Christin Sollerhed, Emma Lilja, Emily Heldt Holmgren, Pernilla Garmy

**Affiliations:** 1 Department of Nursing and Health Sciences, Faculty of Education, Kristianstad University, SE-291 88 Kristianstad, Sweden; ann-christin.sollerhed@hkr.se; 2 Department of Humanities, Faculty of Health Sciences, Kristianstad University, SE-291 88 Kristianstad, Sweden; emmalilja1985@gmail.com (E.L.); kram_emily@hotmail.com (E.H.H.); 3 Department of Health Sciences, Faculty of Medicine, Lund University, SE-221 00 Lund, Sweden

**Keywords:** subjective health, physical activity, body image, body appearance, body functioning, wellbeing in school, body mass index, gender

## Abstract

This study aimed to investigate subjective health and its associations with perceived body image (body appearance and body functioning), physical activity, perceived wellbeing in school, perceived family financial situation, and body mass index among 13- to 15-year-old boys and girls. The study was a cross-sectional study performed in four municipalities in Southern Sweden. Data were obtained from questionnaires completed by adolescents (median age 14; range: 13–15) in Sweden (*n* = 1518, 51% girls), with a participation rate of 73%. Body weight and body height were measured by school nurses and body mass index was calculated. Logistic regression analyses were carried out with subjective health as the dependent variable. Independent variables included in the model were perceived wellbeing in school, perceived family financial situation, perceived body image, physical activity, body mass index, sex, and residency. Variables significantly associated with good subjective health were good wellbeing in school, a perceived good family financial situation, perceived positive body appearance, perceived positive body functioning, being a boy, and high physical activity. Residency and body mass index were not associated with subjective health. Good subjective health is associated with good wellbeing in school, good family financial situation, positive body image, and high physical activity levels. The results highlight the importance of good school climates, the promotion of positive body image, and increased physical activity for adolescents.

## 1. Introduction

Body image consists not only of perceived body appearance, but also of perceived body functioning. Most research has focused on body appearance. However, there is a gap in the literature about the role of perceived body functioning and subjective health [1]. Sollerhed et al. [2] found in a study of school-aged children aged 8–12 that a positive perception of one’s body functioning was associated with a high level of physical fitness, and a recent systematic review found that perceived physical competence is linked with sports attrition [1]. Perceived physical competence is associated with perceived and actual motor skill proficiency [3], which is a global term referring to an individual’s degree of proficiency in performing a wide range of motor skills as well as the mechanisms underlying this performance [4,5,6] and is thus an important factor underlying physical activity in youth. The individual’s ability to develop motor skills proficiency and patterns is linked with body functioning [1,3]. The current study aims to investigate a possible link between subjective health and body image, including body appearance and body functioning. There is also a need for further studies regarding the link between wellbeing in school and subjective health. It is important to investigate subjective health since health is a multi-faceted concept and subjective measures are relevant, especially in efforts for improving health and wellbeing among adolescents. Therefore, this study focuses on subjective health and its associations with perceived body image (body appearance and body functioning), physical activity, perceived wellbeing in school, perceived family financial situation, and body mass index (BMI) among 13- to 15-year-old boys and girls. 

### Background

The health situation among Swedish children and adolescents is generally seen as good. However, subjective health complaints seem to have increased over time [7]. It is also shown, in the latest Health Behavior in School-aged Children (HBSC) study from the WHO, that perceived school stress is increasing among Swedish adolescents, with more adolescents reporting psychosomatic complaints and with the level of physical activity (PA) being very low. Additionally, gender differences in health are presented [8]. An increase in pain and discomfort, as well as an increase in mental health problems, have been shown in Nordic countries, with Sweden having the sharpest increase [9]. An investigation from the Swedish contribution of the HBSC from 1993 to 2017 found that school stress has increased among Swedish school-aged children aged 11–15 during the study period, and school stress has also become more harmful for subjective health [10]. Subjective health has been found to be improved by PA among adolescents [11] and has been shown to be positively correlated with wellbeing and negatively correlated with anxiety and depression among adolescents [12]. Individually customized PA, for at least 30 min and with a frequency of at least three times per week, has been recommended for treating major depressive disorders [13]. PA level has decreased, and sedentary behavior has increased among adolescents over the last decades [14,15]. Among Swedish 13- to 15-year-old adolescents, 10–15% were physically engaging in moderate to vigorous physical activity (MVPA), with girls being the least active [16]. Girls 4–18 years old were shown to perform, on average, 17% less total daily PA than boys [17].

Participation in PA-related behaviors was associated with favorable adolescent health, and high screen-time and sedentary behavior were associated with negative health outcomes [18]. Sedentary behavior includes activities that involve low energy expenditure, with a level of 1.0–1.5 metabolic equivalent units (METs) [19], and is associated with obesity and other negative health consequences [20,21]. Computer, game console, and cell phone use is associated with sedentary behavior, being overweight, and obesity [22,23,24]. In a longitudinal study with more than 250,000 participants from Sweden [25], physical education (PE) class participation in school and additional PA after school hours were shown to be important for subjective health, PA, maximum rate of oxygen consumption (VO2max), and metabolic health in adulthood [25]. 

Exercise and PA are associated with improved body image [26], while a positive body image is a significant positive predictor of MVPA [27]. Body image is defined as the internal, subjective representations of physical appearance and bodily experience that encompasses perception of both body appearance and body functioning. It also has an attitudinal component that reflects how satisfied people are with body appearance and functioning, and involves how a person sees themselves [28]. For young people, thoughts and feelings about appearance and body functioning are vital. Perceived body image and self-perceptions of physical attractiveness have been shown to be correlated with subjective wellbeing [29]. Adolescence is characterized by transition and growth and is a vulnerable period in life [30], and body dissatisfaction often increases during the physical transition from childhood to puberty. Many body function changes are associated with an increase in body mass index (BMI) [31], which in particular affects girls’ body image [32]. Negative perceptions of body weight increase with advancing pubertal status for girls but less for boys [33]. Media representations of appearance ideals are becoming increasingly important in adolescence among both boys and girls [34,35]. The media has a major impact on many adolescents’ perceptions on their body, especially today with lots of time spent on the internet [36]. However, it is not media in itself, but the individual’s internalization of the ideals and adoption as a personal body image standard, which is of importance [37]. 

Based on the assumption that adolescents are influenced by appearance ideals from media and peers at the same time as their body is changing and growing, and subjective health complaints have been shown to increase among Swedish adolescents, the study aimed to investigate subjective health and its associations with perceived body image (body appearance and body functioning), PA level, BMI, perceived wellbeing in school and perceived family financial situation among 13- to 15-year-old boys and girls. The study is relevant for expanding knowledge about the possible link between perceived health, body image, PA, BMI, and school climate. Although the study is conducted in a Swedish setting, the information from the study would also be relevant for students on the same age in other countries and continents. 

## 2. Materials and Methods 

This study was conducted using the quantitative data from a larger research project (ISRCTN17006300). The study was approved by the Regional Ethical Review Board (EPN 2015/113) and conducted in accordance with the Declaration of Helsinki. Participation in the study was voluntary and all of the participants and their parents/legal guardians received both oral and written information about the study. 

The study was a cross-sectional study performed in four municipalities in Southern Sweden. The largest municipality had over 100,000 inhabitants and the smallest had about 15,000. The adolescents were recruited in the 20 schools with grades 7 and 8 from both urban and rural environments, through the school health service. The schools were both public (81%) and private (19%). However, all schools in Sweden are tax funded, and schools are not allowed to have any fees for students. The sample consisted of adolescents aged 13–15 with 49% being boys and 51% girls. The participation rate was 73% (*n* = 1518). Participation was voluntarily, and we did not ask the non-participants for reasons to decline participation. However, the non-participants did not differ regarding gender or age compared with the participants. The response rate of questions in the survey ranged from 98% to 100% and information provided for the calculation of BMI was 91%.

The adolescents were offered an individual visit to the school nurse at the same time that they participated in the survey. The data collection was performed through a questionnaire (see Table 1). The questionnaire included subjective health, PA, body image (perceived body appearance and body functioning), perceived wellbeing in school, perceived physical fitness, perceived family financial situation, age, sex, and residency. The questionnaire has earlier been found valid and reliable among Swedish school-aged children and adolescents [38]. Body weight and body height were measured by the school nurses and body mass index (BMI) was calculated as body weight/body height^2^. The international age and gender-specific BMI cut-off points for children and adolescents were used to define normal weight, being overweight, and obesity [39]. 

### Statistics

The statistics were performed using IBM SPSS Version 24. The data were first analyzed using descriptive statistics with frequencies and percentages. Chi-square tests were used to investigate the associations between subjective health and sex, PA, perceived physical fitness, perceived body appearance and functioning, perceived family financial situation, perceived wellbeing in school, BMI and residency. Subjective health was in earlier studies [40,41,42] found to be affected by gender, socio-economic status and residency. Therefore, these variables were included in the analysis. 

In the crude analysis, the relationship between subjective health and the independent factors wellbeing in school, body appearance, body functions, PA, BMI, sex, socio-economic status and residency was investigated in bivariate analysis. Lastly, a logistic regression was conducted to examine whether wellbeing in school, family financial situation, body appearance, body functions, sex, PA, BMI, and residency had a significant effect on the odds of observing good subjective health. The responses were dichotomized due to few responses in some response options and to facilitate data interpretation. The assumptions of absence of multicollinearity were examined. Variance inflation factors (VIFs) were calculated to detect the presence of multicollinearity between predictors. High VIFs indicate increased effects of multicollinearity in the model. VIFs greater than 5 are cause for concern, whereas VIFs of 10 should be considered the maximum upper limit [43]. McFadden’s R-squared was calculated to examine the model fit, where values greater than 0.2 are indicative of models with excellent fit [44]. The significance level was set at *p* < 0.05. 

**Table 1:** Variables included in the logistic regressions with subjective health as the dependent variable.

Item	Response Options in Questions	Dichotomization
Subjective health “How are you doing most of the time?”	5 categories Very good (1) → Very bad (5)	Very good/Good (1–2) Not so good (3–5) [38]
Perceived wellbeing in school “How do you like school?”	3 categories Very good (1) → Not good at all (3)	Very good (1) Quite good/Not good at all (2–3) [45]
Perceived family financial situation “How well-off do you think your family is?”	5 categories Very good (1) → Very bad (5)	Very good/good (1–2) Not so good (3–5) [45]
Perceived body appearance “How satisfied are you with your body appearance?”	4 categories Completely satisfied (1) → Not at all satisfied (4)	Satisfied (1–2) Not satisfied (3–4) [38]
Perceived body functioning “How satisfied are you with how your body works?”	4 categories Completely satisfied (1) → Not at all satisfied (4)	Satisfied (1–2) Not satisfied (3–4) [38]
Sex	Male/Female	Male Female
Physical Activity “How often do you exercise in your free time for at least half an hour so that you become short of breath and sweaty?”	7 categories Never (1) → 4 times or more (7)	Three times a week or more (6–7) Twice a week or less (1–5) [38]
Body Mass Index	Continuous variable	Cut-off points for children and adolescents to define normal weight, overweight and obesity [46]
Residency	Rural/Urban	Rural Urban

## 3. Results

Characteristics of the full sample are presented in in Table 2. Most adolescents (88%) perceived that their subjective health was “quite good” or “very good”, and 48% of boys and 35% of girls perceived they had very good subjective health. The majority of the adolescents (82%) perceived that their families’ financial situation was “quite good” or “very good”, while 98% reported very good or quite good wellbeing in school, and 61% of boys and 53% of girls reported very good wellbeing in school. Fifty-eight percent reported that they were physically active three or more times per week while 15% were physically active very seldom or were inactive most of the time. About 61% percent of the boys and 56% of the girls were active three or more times per week, while 24% of boys and 29% of girls were active once or twice a week. Most of the adolescents were “completely satisfied” or “quite satisfied” with their body appearance (84%) and body functioning (92%). About 30% of the boys and 18% of the girls were completely satisfied with their body appearance, and 53% of boys and 38% of girls were completely satisfied with body functioning. Most adolescents had normal weight (85%). The prevalence of overweight/obesity was 16% among boys and 15% among girls.

All predictors in the regression model have VIFs less than 10. Table 3 presents the VIF for each predictor in the model.

In Table 4, the crude analysis (bivariate analysis) between subjective health and the independent variables for the unadjusted OR and a 95% CI is presented. In Table 5, the adjusted analysis is presented to investigate the dependent variable subjective health with the independent variables. The model was evaluated based on an alpha of 0.05. The overall model was significant, χ2(8) = 204.26, *p* < 0.001, suggesting that wellbeing in school, family financial situation, body appearance, body functions, sex, PA, BMI, and residency had a significant effect on the odds of observing good subjective health. The McFadden R-squared value calculated for this model was 0.21. The effect of experiencing good wellbeing in school was significant, B = 1.49, OR = 4.44, *p* < 0.001, indicating that experiencing good wellbeing in school increases the odds of having good subjective health by approximately 344% relative to experiencing poor wellbeing in school. The effect of good family financial situation was significant, B = 0.99, OR = 2.68, *p* < 0.001, indicating that good family financial situation increases the odds of good subjective health by approximately 168% relative to poor family financial situation. The effect of being satisfied with body appearance was significant, B = 1.16, OR = 3.19, *p* < 0.001, indicating that being satisfied with body appearance increases the odds of experiencing good subjective health by approximately 219% relative to not being satisfied with body appearance. The effect of being satisfied with body functions was significant, B = 0.81, OR = 2.25, *p* = 0.003, indicating that being satisfied with body functions increases the odds of experiencing good subjective health by approximately 125% relative to not being satisfied with body functions. The effect of being a boy was significant, B = −0.63, OR = 1.89, *p* = 0.002, indicating that being a boy increases the odds of experiencing good subjective health by approximately 89% relative to being a girl. The effect PA (exercising three times a week or more) was significant, B = 0.52, OR = 1.69, *p* = 0.007, indicating that exercising three times a week or more increases the odds of experiencing good subjective health by approximately 69% relative to those exercising less. The effect of being normal weight was not significant, B = −0.10, OR = 0.90, *p* = 0.700, indicating that weight did not have a significant effect on the odds of experiencing good or poor subjective health. The effect of residency was not significant, B = −0.07, OR = 1.07, *p* = 0.749, indicating that living in an urban or rural environment did not have a significant effect on the odds of experiencing good or poor subjective health. Table 5 summarizes the results of the regression model.

## 4. Discussion

In the present study, good subjective health was found to be associated with high wellbeing in school, good family financial situation, positive body image (body appearance and body functioning), high PA level, and male gender. BMI and residence, which was included in the logistic regression model, was not associated with subjective health. 

Adolescents who felt high wellbeing in school were more likely to perceive good subjective health. The school climate has a significant influence on children’s and adolescents’ academic performance and wellbeing. Their experiences in schools not only affect academic development but also influence mental health development, both positively and negatively [47]. The influence of teachers, peers, and learning situations on wellbeing is vital, as is the organizational justice to feel well [48]. Adolescents spend a large share of their time in school and the perceptions of justice and positive psychosocial school climates are important for positive developmental outcomes such as mental health [49]. Adolescents’ health behaviors and views of themselves are related to school [50], and academic stress, relations with teachers and classmates, and noise and disturbance in school are important for perceived health [51]. The environment and climate in schools can predict children’s and adolescents’ health and wellbeing outcomes [52]. Overall, school is important for children and adolescents in both their present and future lives, and therefore the school environment and staff are vital for young people’s health outcomes. School involvement also includes peers and participation in sports activities or other extracurricular activities, which are important for enhanced school bonding and feeling of wellbeing in school [49]. Our study highlights the link between wellbeing in school and subjective health, and therefore calls for action in supporting good study environments for school-aged children and adolescents. 

In our study, adolescents with a perceived good family financial situation were more likely to assess subjective health as being good. The relationship between socioeconomic status (SES) and health is a well-documented finding in social science, though the relationship is less clear since plausible causal mechanisms run in both directions [41]. The design in our study was cross-sectional and therefore does not enable causal explanations. In other studies, subjective socioeconomic status (SES) was associated with self-reported health and it was shown that subjective appraisals of social status could influence health [42]. It is therefore important to include questions about family financial situation in studies concerning health and wellbeing. 

Positive perceived body appearance and positive perceived body functioning of adolescents are strongly associated with a subjective perception of good health. Adolescents who were satisfied with their body appearance and body functioning reported good subjective health more often as peers who were more dissatisfied with their bodies. Those with positive body image rely on their body functioning in different situations, which give them self-esteem and affects their perceived wellbeing [26]. A positive body image is associated with exercise and PA in a reciprocal relation [26,27]. Body image is a multidimensional construct with positive and negative features [53]. Research has focused mostly on understanding its negative features [53] and has provided a rich understanding of negative body image but a rather underdeveloped depiction of positive body image [54]. Body dissatisfaction is implicated in public health concerns, including impaired mental health [55]. As body dissatisfaction was shown to be an important threat to wellbeing [28], it might be interpreted that body satisfaction is a fertilizer for wellbeing. Positive body image is an important prevention and treatment goal for health in young people. Adolescents’ body image is central to their health and wellbeing, which emphasizes the importance of encouraging adolescents to have functional and accepting views of their bodies (for example, through joyful PA and exercise) [56]. 

Sex was associated with subjective health, and a difference between boys’ and girls’ subjective health was found. It was more likely for boys to report good subjective health than it was for girls to do the same, which is in accordance with other studies that have shown gender differences in subjective health in childhood and adolescence [40]. During adolescence, subjective health complaints are more prevalent in girls. The physical transition from childhood to puberty results in less favorable individual attributes such as lower physical fitness and a higher percentage of body fat for girls [31] as well as lower levels of perceived competence in PA and school PE as compared to boys [57]. Low PA among girls is associated with weaker influences from both school and the family environment [57] and PA could be considered part of the intervention and prevention of health complaints among girls [58]. 

Our study found that perceived good subjective health was associated with high levels of PA, which is in accordance with other studies [18,25,59]. More vigorous PA has been shown to have mental benefits beyond moderate PA and associations with fewer health complaints, as well as less stress, pain, and depression [60], and with a positive body image [26]. It has been shown that PA seems to moderate the relationship between school-related stress and health complaints [61]. In particular, MVPA and VO2max are strongly associated with health benefits [62]. The development of the brain and memory performance [63,64] as a result of MVPA is an argument for increased time for PA in both the school setting and in leisure time to increase academic results [65,66]. This, in turn, could augment adolescents’ self-esteem and subjective health [67,68].

As involvement in PA seems to be associated with adolescents’ positive subjective health and body image in several ways, promotion of increased PA among children and adolescents could be a possible way to improve adolescent health, a prescription without negative side effects. Based on the school’s compensatory assignment and the fact that schools encounter children and adolescents with different financial situations, school PE and school health care have a great opportunity to focus on health promotion, encouraging children and adolescents to be physically active and have functional and accepting views of their bodies. This is especially the case with young girls, as girls participate in less MVPA during their leisure time [16,17,69,70] and have a less positive body image. School PE, together with support from school health care, are important in educating children and adolescents to have positive body perceptions, which is a significant positive predictor of MVPA [27]. The perception of one’s body image, especially the body functioning, is important for the ability to do PA, as well as for one’s overall self-perceived physical competence, which is strongly associated with enjoyment (which, in turn, is a major reason for children and adolescents to be physically active) [2,71]. School PE is a special arena for PA, where the focus on the development of young people’s positive body image could be maintained to improve health and quality of life. 

### Strengths and Limitations of the Study

The large sample size representing adolescents from both urban and rural schools is a strength. However, the response rate of 73% has to be considered. A strength is also the objective measure of body weight and body height, measured by trained school nurses. Due to the cross-sectional design, the present study does not enable causal explanations of the empirical results. However, it was shown that involvement in PA was related to positive subjective health, which could be of use in interventions for better health in youth. The results in our study suggest hypotheses about possible explanations of the associations between subjective health and wellbeing in school, body image, PA, and family financial situations. The association between subjective health and gender may be hypothesized to explain the gender differences in health trends. Research questions for future studies might seek to further investigate the perceived school climates and PA levels among adolescent boys and girls. 

A limitation of the study is that all data, except body weight and body height, were collected by questionnaires containing single-item questions. Subjective health was self-reported, as the purpose was to collect data about participants’ own perceptions of their health. Data by questioning young people re valuable, as they can predict objectively measurable health-related outcomes. Therefore, they represent a meaningful indicator of health [72]. PA was self-reported, which is a limitation compared to objectively measured PA. The questionnaire has earlier been found to be reliable and valid among Swedish school-aged children and adolescents [38]. The items were dichotomized in the logistic regression due to few responses in some response alternatives, and to facilitate data interpretation. However, dichotomization can increase the risk of overlooking nuances in the results. In the current study, no association between BMI and subjective health was found. The great majority of the participants (85%) had normal weight, and therefore weight issues might be a minor problem for most of them. 

## 5. Conclusions

Good subjective health is associated with good wellbeing in school, a good family financial situation, positive body image, and high PA levels. The results highlight the importance of good school climates and promotion of positive body image, with a special focus on body functioning and increased PA for adolescents. Increased involvement in PA as it is important for overall health in children and adolescents should be promoted in school through the school PE, school health care, and in general through society.

## Figures and Tables

**Table 2:** Bivariate analysis of subjective health and other factors among Swedish adolescents aged 13–15 years (*n* = 1518).

	Good Subjective Health (%)	Poor Subjective Health (%)	*p*-Value *
Sex ^a^			
Girls	632 (48.0)	126 (70.0)	<0.0001
Boys	685 (52.0)	54 (30.0)	
Age ^a^			
13 years	174 (13.1)	16 (8.8)	0.191
14 years	1138 (85.9)	163 (89.6)	
15 years	13 (1.0)	3 (1.6)	
Perceived wellbeing in school ^a^			
Very good	814 (61.8)	37 (20.6)	<0.0001
Quite good	489 (37.1)	127 (70.6)	
Not good at all	14 (1.1)	16 (8.9)	
Perceived family financial situation ^a^			
Very good	640 (48.9)	47 (26.4)	<0.0001
Quite good	463 (35.4)	70 (39.3)	
Average	190 (13.6)	50 (28.1)	
Not so good	12 (0.9)	7 (3.9)	
Not good at all	2 (0.2)	4 (2.2)	
Perceived body appearance ^a^			
Yes, completely satisfied	347 (26.3)	12 (6.7)	<0.0001
Yes, quite satisfied	810 (61.4)	87 (48.3)	
No, quite dissatisfied	143 (10.8)	59 (32.8)	
No, not at all satisfied	19 (1.4)	21 (11.7)	
Perceived body functioning ^a^			
Yes, completely satisfied	654 (49.5)	28 (15.6)	<0.0001
Yes, quite satisfied	596 (45.1)	105 (58.3)	
No, quite dissatisfied	69 (5.2)	41 (22.8)	
No, not at all satisfied	2 (.2)	6 (3.3)	
Physical activity (PA) ^a^			
Never	42 (3.2)	8 (4.4)	<0.0001
Sometime per year	30 (2.3)	9 (5.0)	
Sometime per month	105 (7.9)	30 (16.6)	
Regularly once a week	128 (9.7)	23 (12.7)	
Regularly twice a week	213 (16.1)	39 (21.5)	
Regularly three times a week	305 (23.1)	34 (18.8)	
Regularly four times or more	500 (37.8)	38 (21.0)	
BMI ^b^			
Normal weight	1043 (85.4)	128 (79.0)	0.037
Overweight/obesity	178 (14.6)	34 (21.0)	
Residence ^a^			
Urban	945 (71.3)	128 (70.3)	0.794
Rural	380 (28.7)	54 (29.7)	

* Chi Square. Missing values: **^a^** < 2.1%; **^b^** = 8.9%.

**Table 3:** Variance Inflation Factors for wellbeing in school, family financial situation, body appearance, body functions, sex, PA, BMI, and residency (*n* = 1518).

Variable	VIF
Wellbeing in school	1.02
Family financial situation	1.03
Body appearance	1.16
Body functions	1.14
Sex	1.04
PA	1.02
BMI	1.14
Residency	1.03

**Table 4:** Crude analysis of the associations between good subjective health and independent variables among Swedish adolescents aged 13–15 years (*n* = 1518).

Variable	*B*	*SE*	χ^2^	*p*	*OR*	95% CI
(Intercept)	1.26	0.09	176.14	<0.001	-	-
Good wellbeing in school *	1.83	0.19	90.28	<0.001	6.25	[4.29, 9.13]
(Intercept)	1.21	0.15	68.44	<0.001	-	-
Good family financial situation *	1.04	0.18	34.99	<0.001	2.82	[2.00, 3.98]
(Intercept)	0.71	0.14	26.66	<0.001	-	-
Satisfied with body appearance *	1.75	0.17	103.67	<0.001	5.77	[4.12, 8.09]
(Intercept)	0.41	0.19	4.81	0.028	-	-
Satisfied with body functions *	1.83	0.21	76.50	<0.001	6.22	[4.13, 9.37]
(Intercept)	1.61	0.10	273.20	<0.001	-	-
Sex (Boy) *	0.93	0.17	29.18	<0.001	2.53	[1.81, 3.54]
(Intercept)	1.56	0.11	218.76	<0.001	-	-
PA (Three times a week or more) *	0.86	0.16	27.90	<0.001	2.35	[1.71, 3.23]
(Intercept)	1.66	0.19	78.23	<0.001	-	-
BMI (Normal weight) *	0.44	0.21	4.47	0.035	1.56	[1.03, 2.35]
(Intercept)	2.00	0.09	450.55	<0.001	-	-
Residency (Rural) *	−0.05	0.17	0.08	0.782	0.95	[0.68, 1.34]

* Note. Good wellbeing in school: χ^2^(1) = 112.63, *p* < 0.001, McFadden *R*^2^ = 0.10. * Note. Good family financial situation: χ^2^(1) = 32.09, *p* < 0.001, McFadden *R*^2^ = 0.03. * Note. Satisfied with body appearance: χ^2^(1) = 96.12, *p* < 0.001, McFadden *R*^2^ = 0.09. * Note. Satisfied with body functions: χ^2^(1) = 66.70, *p* < 0.001, McFadden *R*^2^ = 0.06. * Note. Sex (Boy): χ^2^(1) = 31.51, *p* < 0.001, McFadden *R*^2^ = 0.03. * Note. PA (Three times a week or more): χ^2^(1) = 28.62, *p* < 0.001, McFadden *R*^2^ = 0.03. * Note. BMI (Normal weight): χ^2^(1) = 4.20, *p* = 0.041, McFadden *R*^2^ = 0.00. * Note. Residency (Rural): χ^2^(1) = 0.08, *p* = 0.782, McFadden *R*^2^ = 0.00.

**Table 5:** Logistic regression analysis of factors related to subjective health (good or very good) among Swedish adolescents aged 13–15 years adjusted by gender, economic status and residency (*n* = 1518).

Variable	*B*	*SE*	χ^2^	*p*	*OR*	95% CI
(Intercept)	−1.33	0.35	14.65	< 0.001	-	-
Good wellbeing in school	1.49	0.22	46.36	< 0.001	4.44	[2.89, 6.81]
Good family financial situation	0.99	0.21	21.99	< 0.001	2.68	[1.77, 4.04]
Satisfied with body appearance	1.16	0.22	27.93	< 0.001	3.19	[2.07, 4.90]
Satisfied with body functions	0.81	0.28	8.62	0.003	2.25	[1.31, 3.87]
Sex (Boy)	0.63	0.20	9.63	0.002	1.89	[1.26, 2.81]
PA (Three times a week or more)	0.52	0.19	7.27	0.007	1.69	[1.15, 2.47]
BMI (Normal weight)	−0.10	0.26	0.15	0.700	0.90	[0.54, 1.51]
Residency (Rural)	−0.07	0.22	0.10	0.749	1.07	[0.70, 1.65]

Note. χ2(8) = 204.26, *p* < 0.001, McFadden R2 = 0.21. OR, Odds ratio; CI, Confidence interval.

## Data Availability

The data presented in this study are available on request from the corresponding author. The data are not publicly available due to restrictions from the ethical review board.

## Abbreviations

BMI	Body Mass Index
HBSC	Health Behavior in School-aged Children
PA	Physical Activity
PE	Physical Education
